# SVM-Based Synthetic Fingerprint Discrimination Algorithm and Quantitative Optimization Strategy

**DOI:** 10.1371/journal.pone.0111099

**Published:** 2014-10-27

**Authors:** Suhang Chen, Sheng Chang, Qijun Huang, Jin He, Hao Wang, Qiangui Huang

**Affiliations:** School of Physics and Technology, Wuhan University, Wuhan, China; Xiamen University, China

## Abstract

Synthetic fingerprints are a potential threat to automatic fingerprint identification systems (AFISs). In this paper, we propose an algorithm to discriminate synthetic fingerprints from real ones. First, four typical characteristic factors—the ridge distance features, global gray features, frequency feature and Harris Corner feature—are extracted. Then, a support vector machine (SVM) is used to distinguish synthetic fingerprints from real fingerprints. The experiments demonstrate that this method can achieve a recognition accuracy rate of over 98% for two discrete synthetic fingerprint databases as well as a mixed database. Furthermore, a performance factor that can evaluate the SVM's accuracy and efficiency is presented, and a quantitative optimization strategy is established for the first time. After the optimization of our synthetic fingerprint discrimination task, the polynomial kernel with a training sample proportion of 5% is the optimized value when the minimum accuracy requirement is 95%. The radial basis function (RBF) kernel with a training sample proportion of 15% is a more suitable choice when the minimum accuracy requirement is 98%.

## Introduction

Fingerprint recognition is a relatively mature biometric identification [1], [2] method, and automatic fingerprint identification systems (AFISs) have been widely used throughout our lives. However, because AFISs normally connect with interests, attacks on such systems are ongoing. N.K. Ratha proposed a biometric system with eight possible attack points [3]. Providing a fake fingerprint image is a particularly simple and effective method of fraud that poses a considerable threat to the security of AFISs.

There are three typical fake fingerprints: altered fingerprints, non-living fingerprints and synthetic fingerprints. Altering a fingerprint directly changes the texture of the finger by obliteration, distortion and imitation. A.K. Jain et al. published in-depth research about this topic, including curvature histogram analysis [4], a minutiae-step algorithm [5] and ‘Z’-cut restoration [6]. However, because altering a fingerprint causes irreversible damage to the finger, it is not widely used except by criminals.

The use of a non-living fingerprint avoids this negative side effect. A non-living fingerprint is made by transferring the texture of the finger onto another material, such as Play-Doh, latex rubber, silicone, latex paint or plastic. In this manner, fingerprint information is separated from individuals, and the corresponding relationship between them is destroyed. Physiological information capture is a method of recognizing a non-living fingerprint because the fake fingerprint loses the finger's physiological characteristics, such as temperature, pulse oximetry or ECG signal [7]. Image-based discrimination is more commonly used because it does not require special hardware. R. Derakhshani et al. captured fingerprint images in 0 s and 5 s and detected the change in skin perspiration pattern features [8]. A. Antonelli et al. analyzed the fingerprint distortion after capturing fingerprint images continuously [9]. J. Galbally et al. [10] and L.F.A. Pereira et al. [11] extracted characteristic factors and used classifiers to distinguish non-living fingerprints.

A synthetic fingerprint is different from the above two types of fingerprints in that it is entirely false. For example, D. Kosz [12] designed an algorithm that employs a proprietary mathematical model of finger ridge patterns to synthesize a fingerprint. R. Cappelli et al. proposed a five-step method to generate synthetic fingerprints in 2000 [13] and then added wet/dry fingerprinting [14] and Perlin Noise [15] to improve its quality, finally developing a synthetic fingerprint database, FVC2004 DB4 [16], which was successfully used in the International Fingerprint Verification Competition [17]. Furthermore, J. Hu et al. improved this algorithm by improving the orientation field model, density map model and ridge texture model, which made the synthetic fingerprint look more realistic [18], and then developed a synthetic fingerprint generator software program, FPGenerator [19], which was used in the China Biometric Verification Competition. Another type of algorithm is called fingerprint reconstruction [20], [21], which restores a fingerprint image from the fingerprint's minutiae. In contrast to fingerprint synthesis, the goal of fingerprint reconstruction is to obtain a fingerprint that is as close as possible to the original fingerprint. The minutiae from an existing fingerprint must be provided, whereas no input is needed for fingerprint synthesis. However, certain fingerprint reconstructions, such as that by Q. Zhao et al. [22], use statistical feature models to provide singular points, orientation field and minutiae, which is different from traditional fingerprint reconstruction in terms of the generation of minutiae and can be treated as a synthetic fingerprint.

Because synthetic fingerprints have been successfully used in competitions to evaluate fingerprint matching algorithms, it is reasonable to infer that synthetic fingerprints can deceive AFISs, which is a considerable potential threat to the security of fingerprint-based application systems. Unfortunately, to the best of our knowledge, no method has previously been developed to discriminate synthetic fingerprints from real ones.

To solve this problem, an effective algorithm that can discriminate synthetic fingerprints from real fingerprints is proposed in this paper. Six typical features of synthetic fingerprints are extracted and combined using a support vector machine (SVM) classifier. The algorithm is verified on a mixed database composed of real fingerprints from FVC2004 DB2 [23] and synthetic fingerprints from FVC2004 DB4 and FPGenerator. The experimental results demonstrate that the accuracy of our algorithm exceeds 98%, successfully discriminating synthetic fingerprints from real ones. Furthermore, the effect of the number of training samples and different kernel functions on the SVM is studied in depth, and a performance factor that can quantitatively evaluate the SVM's performance is presented.

The remainder of this paper is organized as follows. The feature extraction algorithm is described in Section 2. Section 3 presents the discrimination results based on the SVM. Section 4 discusses the quantitative optimization strategy of SVM intelligent computing. The conclusions are presented in section 5.

## Methods

**Figure 1:**
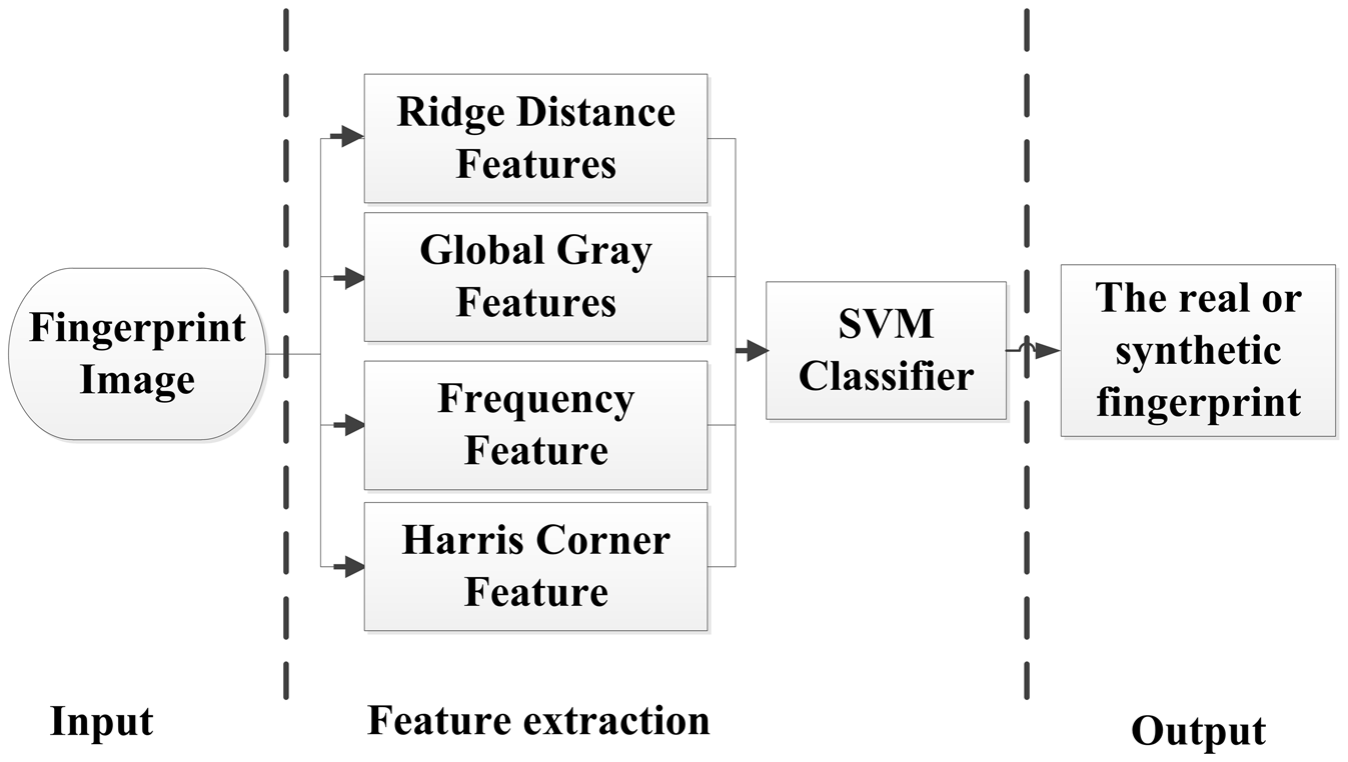
Structure of our synthetic fingerprint discrimination.

The proposed discrimination algorithm consists of synthetic fingerprint feature extraction and SVM classification, as shown in Fig. 1. The ridge distance average feature and ridge distance standard deviation feature (collectively called the ridge distance features in Fig. 1), the global gray average feature and the global gray variance feature (collectively called global gray features in Fig. 1), the frequency feature and the Harris Corner feature are extracted from the image to constitute the feature vector, as defined in (1), in which *InputMatrix* is the input matrix of the SVM, *V_m_* is the *m*th fingerprint image's feature vector, and *Q_mn_* is the *m*th fingerprint image's *n*th feature factor. Here, *n* = 6, as six features are extracted. The feature extraction algorithm is detailed below:
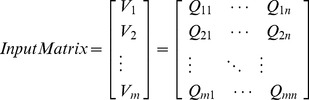
(1)


### Ridge Distance Features

The structure of the ridge and valley lines is one of the most obvious characteristics of a fingerprint. For a synthetic fingerprint, the ridge is normally generated by fingerprint image enhancement, such as the Gabor filter [13] in FVC2004 DB4. However, in this case, a major disadvantage is that the ridge width is equal to the valley width [18]. In FPGenerator, the algorithm is improved by constructing filter functions with cosine functions of different periods [18]. Thus, the ridge width is not equal to the valley width, but the sum of the width of the ridge and valley (typically called the ridge distance [24]) remains unchanged. In contrast, the ridge width of a real fingerprint is not always equal to the valley width, and the distance will change from person to person. Thus, the average ridge distance and standard deviation are selected to calculate the ridge distance and relative variation.

**Figure 2:**
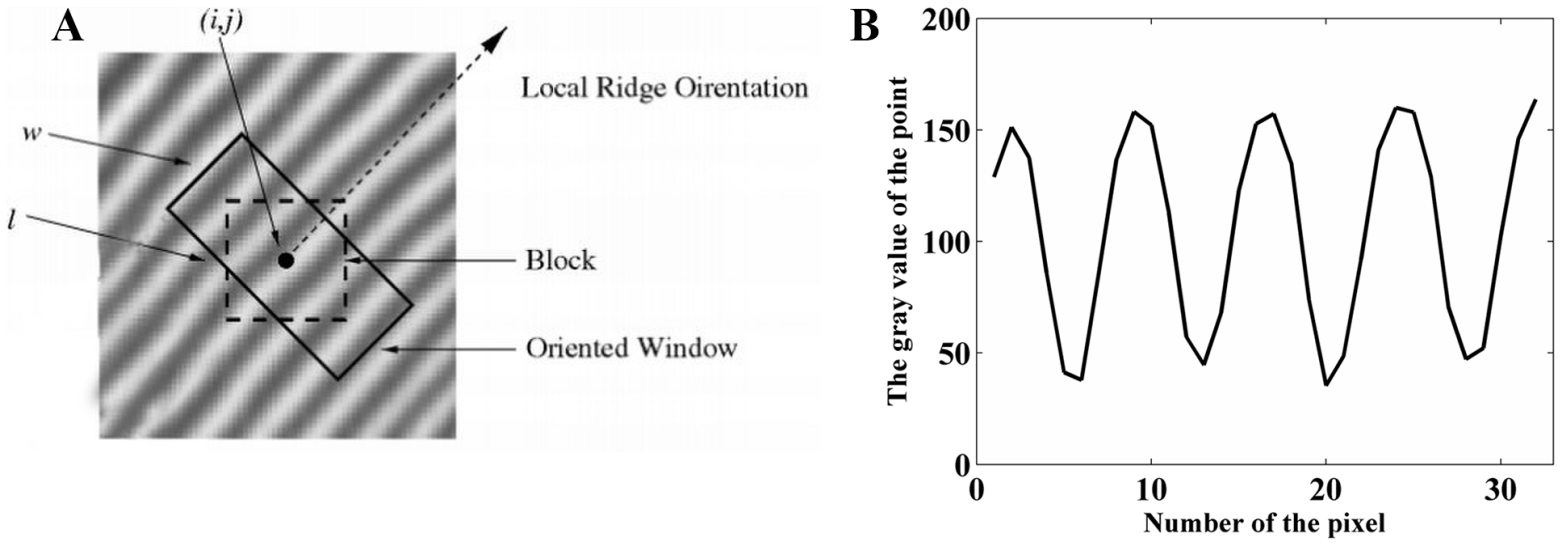
The window-based ridge distance calculation. (A) Window in a fingerprint, (B) gray arrary curve of ridge distance.

To obtain ridge distance features, a rectangular window (32×16) perpendicular to the ridge line direction is established, as shown in Fig. 2(a). There are 16 pixel gray values in each column along the direction perpendicular to the ridge, and the average value of these 16 pixels can be calculated. Here, *f(u,v)* is the gray of point *(u,v)*. There are 32 average gray values in this window, and they can be combined as a gray array *S[k]*:
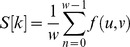
(2)

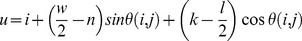
(3)

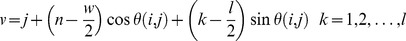
(4)where *l* and *w* are the length and width of the rectangular window (*l* = 32, *w* = 16), respectively, 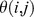 is the direction of the point 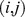 in the fingerprint, and *n* 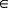 [0,15] labels the 16 pixels in each column. *S[k]* is a type of sine wave, as shown in Fig. 2(b), and the number of pixels between two peak values is the ridge distance.

To improve the algorithm's accuracy, a 96 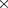 96 block area is chosen from the center of the fingerprint's foreground. As calculated by (5) and (6), *Q_disavg_* and *Q_disstd_* are the average and standard deviation of these 9,216 values, respectively. *D_n_* is the ridge distance of the *n*th point, and the value is the number of pixels between two peaks in the *S[k]*'s sine wave image.
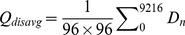
(5)

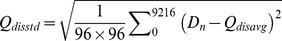
(6)


**Figure 3:**
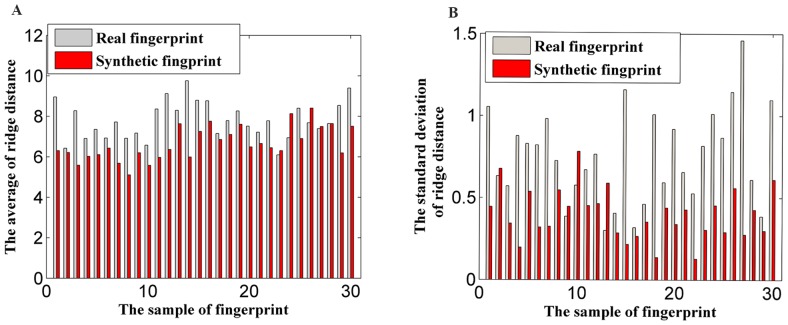
Typical data distribution of ridge distance features. (A) Distribution of *Q_disavg_*, (B) distribution of *Q_disstd_*.

The effect of these two features is verified in Fig. 3. The average ridge distance and standard deviation of real fingerprints are typically larger than those of synthetic ones, which indicate that these two features are suitable discrimination factors. Only some typical samples (30 DB2 real fingerprints, 15 DB4 synthetic fingerprints, and 15 FPGenerator synthetic fingerprints) from the 1,200 tested fingerprint images are shown for clarity.

### Global Gray Features

**Figure 4:**
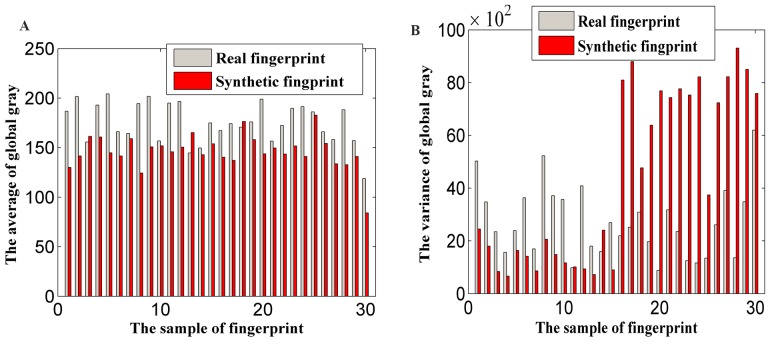
Typical data distribution of global gray features. (A) Distribution of *Q_grayavg_*, (b) distribution of *Q_grayvar_*.

This study also focuses on the background of the fingerprint image. The optical background is analyzed in this paper because the FVC2004 DB4 fingerprint images are optical backgrounds; such a background uses a statistical model algorithm based on Karhunen-Loeve Transform (KLT) training [18]. We investigate the difference between the KLT training background and the real background based on the gray-scale aspect. The global gray average and variance of the entire image are as follows:
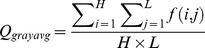
(7)

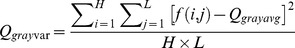
(8)where *Q_grayavg_* is the global gray average, *Q_grayvar_* is the global gray variance, 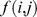 is the gray of point 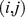, and the size of the image is *H* 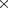 *L*. Fig. 4 displays the effect of the global gray features and demonstrates that the gray average values of synthetic fingerprints are less than those of the real ones, and parts of their gray variance (samples from FPGenerator) are greater than the real ones. Overall, the real fingerprints and the two types of fake fingerprints differ from one another in terms of their global gray features.

### Frequency Feature

**Figure 5:**
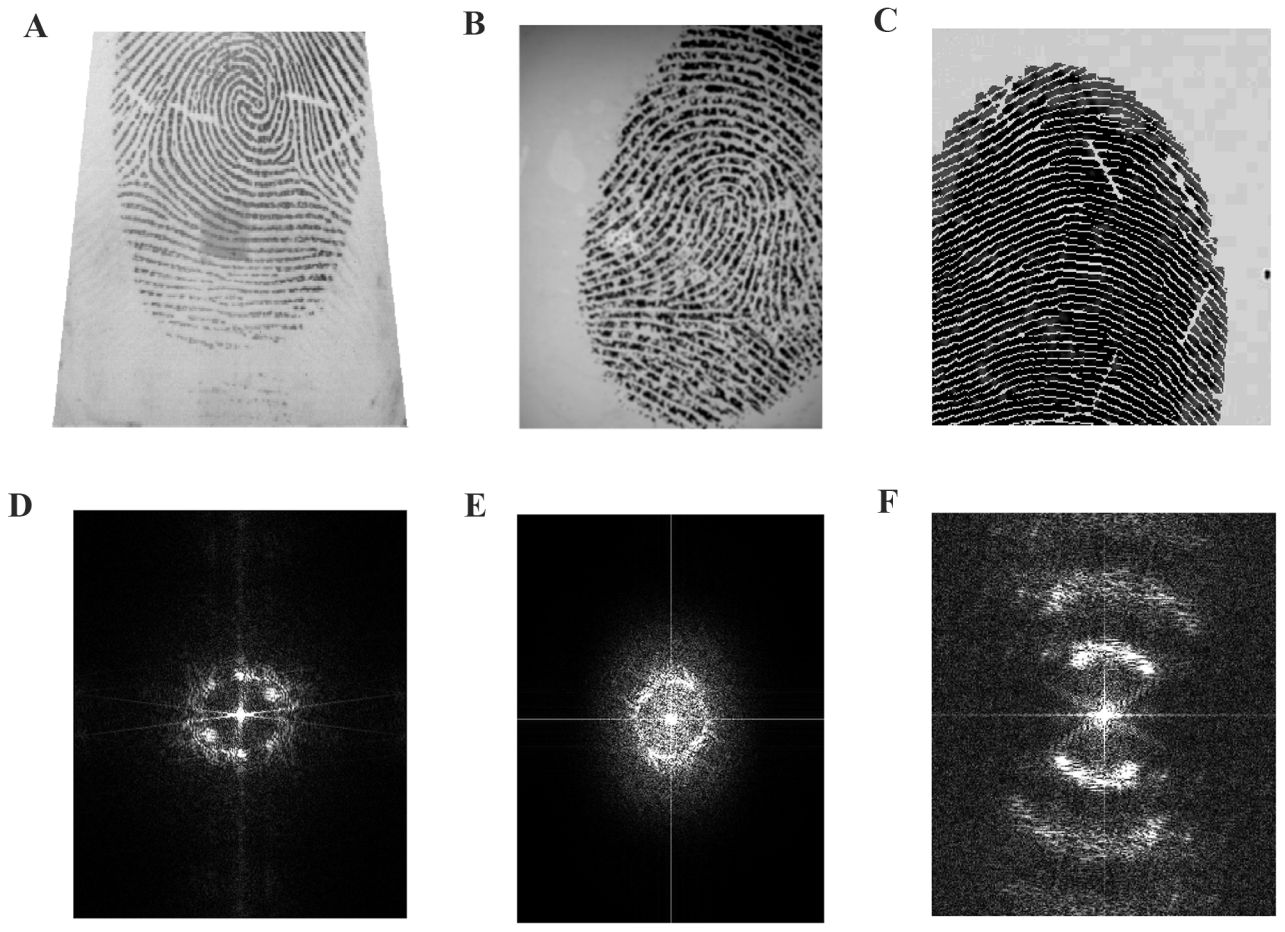
Fingerprint's frequency analysis. (A) A real fingerprint, (B) a DB4 fingerprint, (C) a FPGenerator fingerprint, (D) (A)'s DFT analysis, (E) (B)'s DFT analysis, (F) (C)'s DFT analysis.

Noise is another characteristic that we used to distinguish synthetic fingerprints. When a real fingerprint is captured, its noise is expressed as a small white Gaussian noise. However, a synthetic fingerprint exhibits a relatively large amount of noise. Fig. 5 presents the discrete Fourier transform (DFT) of fingerprints, in which the bright, discrete points out of the concentrated ring represent high-frequency noise energy in the fingerprint images. The pattern of a real fingerprint is similar to the pattern of a DB4 fingerprint; however, these two patterns clearly differ from the pattern of an FPGenerator fingerprint, which is distributed across a large area and has clearly discrete energy.

**Figure 6:**
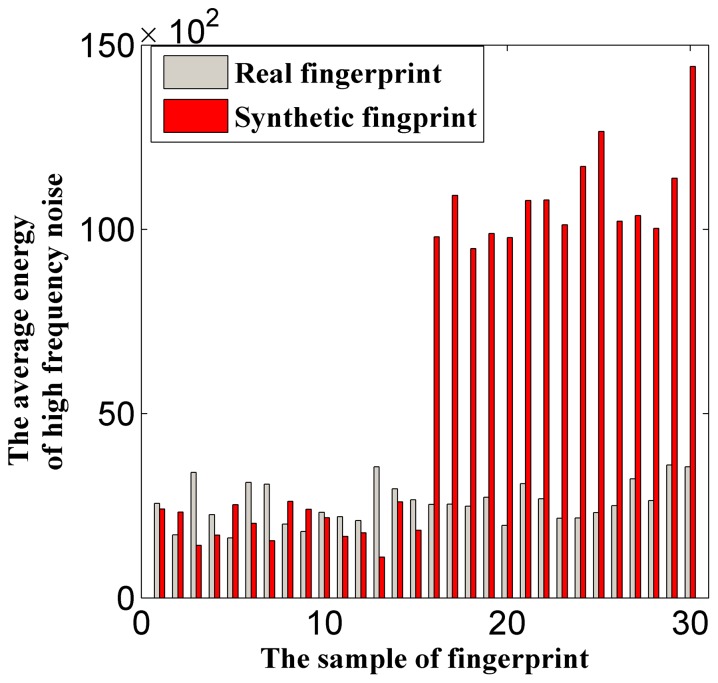
Data distribution of frequency feature.

To apply this frequency characteristic, we calculate the average value of high-frequency noise energy outside the central bright ring in polar coordinates using (9): 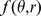 represents the point on the image converted into polar coordinates, and r 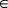 (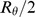, 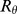], 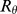  = *min(H,L)*. *L* and *H* are the length and width of the image, respectively. Fig. 6 presents the data distribution of the frequency features. The Q*_fft_* value of the FPGenerator fingerprint (the last 15 samples of synthetic fingerprints in Fig. 6) is clearly different from those of the other fingerprints, which is in accordance with Fig. 5.


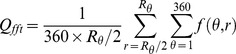
(9)

### Harris Corner Feature

Small white blobs are often added to simulate a finger's noise, which makes synthetic fingerprints less smooth than real ones at the minutia scale. Based on this characteristic, a texture evaluation method, Harris Corner [25], is introduced to our algorithm as follows.
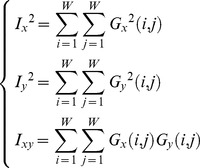
(10)

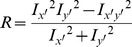
(11)


The fingerprint image is divided into *W* 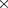 *W* (*W* = 8) small blocks. *G_x_*(*i,j*) and *G_y_*(*i,j*) are the gradient values in the horizontal and vertical directions, respectively, calculated by the Sobel operator at the point 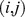. *I_x'_, I_y'_* and *I_x'y'_* are the Gaussian smoothing filter results of *I_x_, I_y_* and *I_xy_*, respectively, to reduce noise. The corner R is calculated based on these values. If R is the local maximum or is larger than a threshold (set at 5,500 in our algorithm), R is considered a Harris Corner. The number of Harris Corners is our Harris Corner feature, *Q_harris_*.

**Figure 7:**
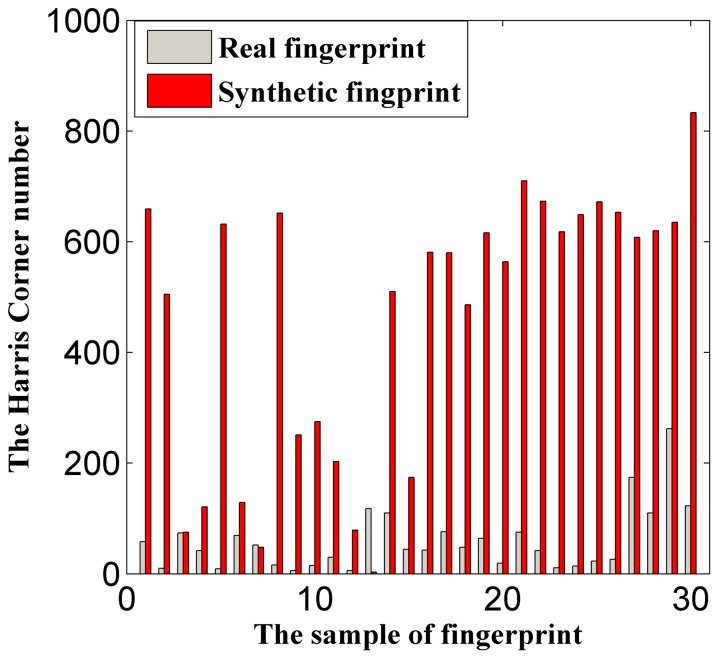
Data distribution of Harris Corner feature.

Fig. 7 indicates a notable difference between real fingerprints and the two types of synthetic fingerprints; namely, the real fingerprint images are smoother. Thus, the Harris Corner feature can be used to discriminate synthetic fingerprints.

## Results

After the above features are extracted, a classifier is needed to discriminate synthetic fingerprints from real ones. Among mainstream intelligent classifiers, we chose the SVM [26] because it performs well and requires relatively few training samples [27]. To analyze the ability of a SVM to recognize synthetic fingerprints, the three most common SVM kernel functions, linear, polynomial and RBF, were tested. Their formulations are as follows:
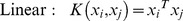
(12)

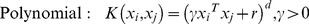
(13)

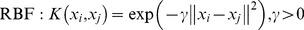
(14)where γ, *r* and *d* are parameters of the kernel function [28].

These three kernel functions are used in a classifier, LibSVM [29], with characteristic vectors, which consist of the six characteristic factors extracted in section 3. These vectors are expressed as follows:
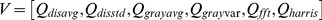
(15)


### Database and Workbench

In this paper, the entire sample database consists of 1,200 fingerprint images. Six hundred real fingerprint images are chosen randomly from FVC2004 DB2. Three hundred synthetic fingerprints are taken from FVC2004 DB4, and the remaining three hundred synthetic fingerprints are generated by the FPGenerator software. Of course, there are other synthesis methods, but to our knowledge, no public database is available. As the fundamental principles of most synthetic fingerprints are similar, it is reasonable to infer that our proposed method can be applied to most synthetic fingerprints. For example, Q. Zhao et al. [22] used statistical feature models and a reconstruction algorithm [21] to obtain a binary synthetic fingerprint image. The noising and rendering algorithm used in that paper are the same as those in FVC2004 DB4, which is included in our test.

The software workbench is MATLAB2010a under Windows 7. The hardware workbench is Intel T6570 2.1 GHz CPU, 2G memory. The SVM classifier is LibSVM (Ver. 2.89-3) [30].

### Experimental Results

**Figure 8:**
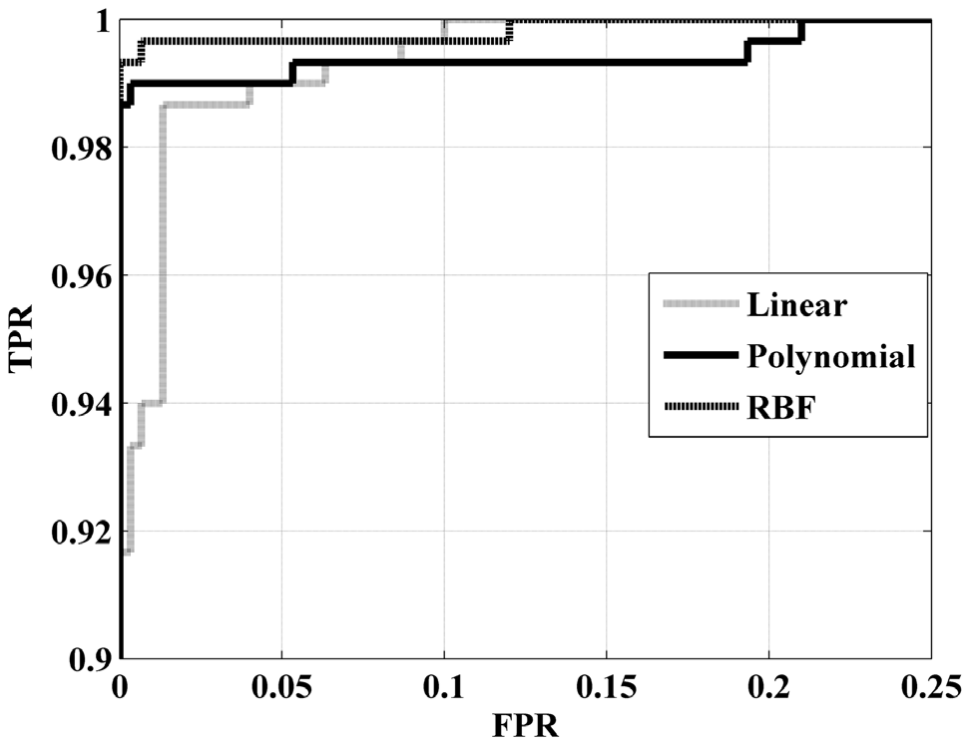
ROC curves of different kernel functions on the mixed database.

To verify our method's versatility, in addition to the recognition of two types of synthetic fingerprints, discrimination is tested on the mixed database, which is composed of 300 FVC2004 DB4 synthetic fingerprints and 300 FPGenerator synthetic fingerprints. Table 1 illustrates that even in the mixed database, our algorithm's identification accuracy exceeds 98% for all three kernel functions. In this case, the results are the average of 1,000 repetitions to reduce the effect of random error. The results prove that our feature factors have strong robustness. The ROC curve in Fig. 8 supports this result. To generalize our experiments, all of the following tests are based on the mixed database.

**Table 1:** The accuracy rate of different synthetic databases on different kernel functions.

Database	Linear (%)	Polynomial (%)	RBF (%)
FCV2004 DB4	99.87	99.68	99.77
FPGenerator	99.94	99.85	99.38
Mixed Database	98.67	99.17	99.17

Table 2 presents a detailed comparison of the kernel functions. Although they have approximately similar accuracy, their training and testing times differ remarkably. The polynomial method is the fastest, whereas the RBF method has a relatively long testing time and the linear method has an extremely long training time. Although the absolute value of the processing times is small in this case, it is critical to compare the kernel functions' efficiency, especially for the discrimination of massive fingerprints or for embedded applications.

**Table 2:** The comparison between different kernel functions.

Kernel function	Linear	Polynomial	RBF
Accuracy rate (%)	98.67	99.17	99.17
Training time (ms)	691.63	70.80	97.99
Testing time (ms)	1.47	1.53	8.15
AUC	0.9983	0.9985	0.9996

## Discussion

The traditional discussion of fingerprint recognition is presented in section 3, but the results in Table 2 are inadequate for evaluating the performance of the SVM. The choice of a suitable kernel function for the SVM is typically based on experience and trial. To solve this problem, we discuss the performances of typical SVM kernel functions and propose a quantitative performance factor for the optimal design of intelligent computing.

**Figure 9:**
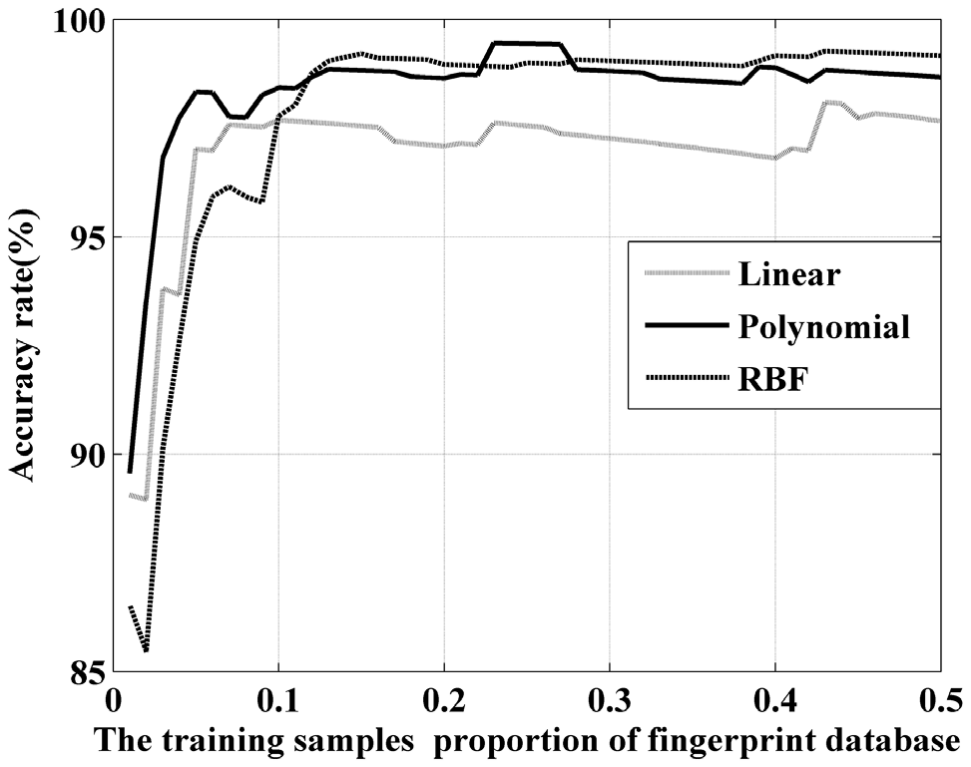
The relationship between training samples proportion and discrimination accuracy on different kernel functions.

In traditional SVM training, the proportion of training and testing samples is 1∶1. As the number of training samples influences the accuracy rate and training time considerably, we believe that 1∶1 is not the optimal configuration of the SVM computing. To test this belief, Fig. 9 illustrates the relationship between the training sample proportion and discrimination accuracy. The accuracies of all three kernels exhibit an increasing trend with the sample proportion, but they saturate before a training proportion of 50%. When the accuracy rate is stable, the polynomial and RBF kernel have a higher accuracy than the linear kernel. The polynomial is superior when the training sample proportion is small, whereas the RBF is the optimal kernel when the proportion is high. This situation illustrates that the training sample proportion should be optimized.

The efficiency of kernel functions is presented in Table 3. The training times are the average of 1,000 computations to eliminate volatility error. The training time increases with an increasing number of training samples. The linear method has a particularly long training time, and the polynomial method is slightly superior to the RBF method.

**Table 3:** The training time of different training samples number.

Number of training samples	Training time of Linear (s)	Training time of Polynomial (s)	Training time of RBF (s)
1200	4.261	0.143	0.180
600	0.693	0.065	0.081
300	0.168	0.030	0.031
50	0.002	0.001	0.003

Based on the above analysis, we suggest a quantitative performance factor to evaluate the accuracy and efficiency of the SVM. Because the accuracy and training time must be balanced, we set a minimum accuracy that must be achieved in application. For applications demanding a higher level of accuracy, the performance of the SVM can be assessed as follows:
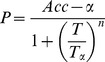
(16)where 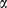 is the minimum accuracy and *Acc* is the recognition accuracy rate under a training sample proportion. *T* is the training time, 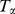 is the training time for the minimum level of accuracy, and *n* (*n* 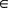 (0,1]) is a parameter that represents the sensitivity of the training time. A larger *n* indicates a shorter duration. Because the testing time is not sufficiently long compared with the training time, the latter is used to represent the efficiency of the SVM in this paper.

**Figure 10:**
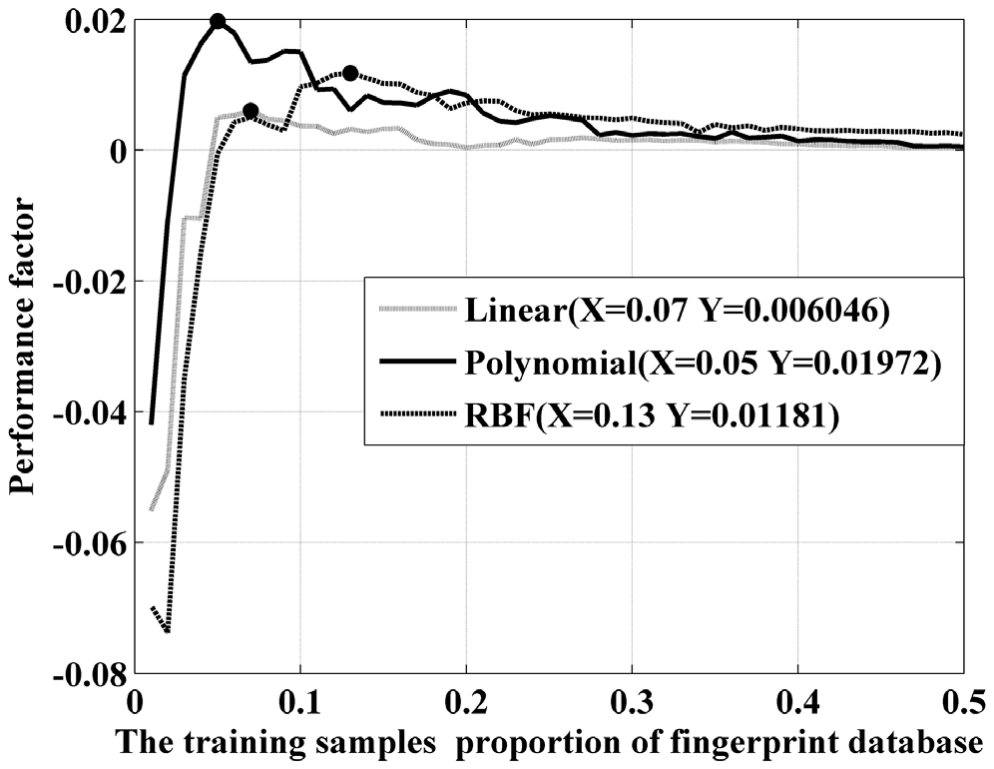
Performance factor of different kernel functions on 95% standard accuracy.

The P values of these three kernel functions are shown in Fig. 10. We test these functions using the database of 1,200 mixed fingerprint images and set the sensitivity parameter to 1 and the minimum accuracy 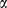 to 95%, which is sufficient for most applications. The 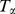 of the linear kernel, polynomial kernel and RBF kernel are 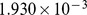, 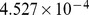 and 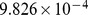 s, respectively. The peak values of the three performance factors are our optimized design points, and their coordinates are labeled.

The polynomial kernel peaks first at a training sample proportion of 5%, whereas the linear kernel and RBF kernel require more samples to reach their peaks. A negative P value indicates that the discrimination does not reach the minimum accuracy and should not be considered. Based on the curve of our performance factor, we can quantitatively state that the polynomial kernel function with a training sample proportion of 5% is the optimal configuration in this case.

The sensitivity parameter is tested in Table 4. Here, *n* = 0.1 denotes the extreme condition in which the training time is nearly not considered. In this case, the optimized accuracies of all three kernels increase slightly, but the training times increase accordingly. For the linear kernel, the training time increases by approximately 1,300% with only a 0.5% improvement in accuracy. The polynomial kernel also exhibits a relatively balanced performance, and the RBF kernel exhibits good stability in terms of training time control. If the size of the test database increases, the training times will increase sharply, and the gap between different kernels will be more apparent.

**Table 4:** Three kernel function's performance under different sensitive parameters.

Optimized Performance	Linear	Polynomial	RBF
Accuracy rate when n = 1 (%)	97.58	98.33	99.04
Accuracy rate when n = 0.1 (%)	98.10	99.46	99.22
Training time when n = 1 (ms)	6.60	0.35	2.30
Training time when n = 0.1 (ms)	85.50	3.20	3.00

**Figure 11:**
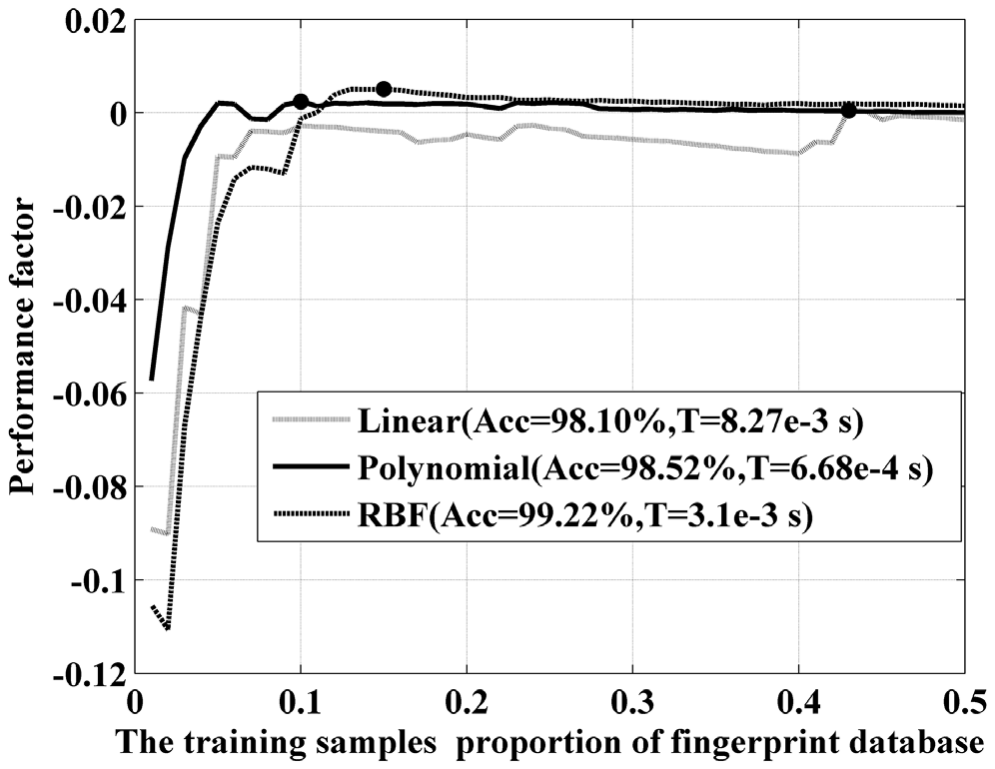
Performance factor of different kernel functions on 98% standard accuracy.

Finally, we consider the opposite extreme condition, in which the minimum discrimination accuracy is increased to 98% (while the sensitive parameter is still 1). The results are shown in Fig. 11. Under this higher accuracy standard, all three kernel functions require a higher training sample proportion. The bulk of the linear kernel's curve indicates that this method is not suitable for this high standard. The polynomial kernel still requires fewer training samples to achieve the desired recognition accuracy. The RBF kernel has the highest peak value, demonstrating that it is the optimal choice. Furthermore, if the accuracy standard continues to rise, the RBF kernel function will be the only function that can satisfy the requirements.

The above discussion can be used to determine a quantitative optimization strategy for SVM fingerprint recognition. Under the minimum accuracy required by the application, the P factor of the candidate kernels with varying training sample proportions should be calculated. The highest peak point of those performance factor curves is the optimized configuration. In our case, the polynomial kernel with a training sample proportion of 5% is the optimal value when the accuracy requirement is normal (95%), whereas the RBF kernel with a training sample proportion of 15% is a better choice when the accuracy requirement is extremely high (98%).

## Conclusions

This paper proposes an effective algorithm for synthetic fingerprint discrimination and a quantitative optimization strategy. Six specific characteristic features are extracted, and a SVM method is used to discriminate synthetic fingerprints. The method can achieve a recognition accuracy exceeding 98% for two types of synthetic fingerprints separately as well as for mixed cases. More importantly, a performance factor for SVM classification optimization is defined. Based on this performance factor, a quantitative optimization strategy is established, which can yield the optimal values of the SVM kernel function and training sample proportion. This method overcomes the dilemma of experience-based parameter selection and can also guide intelligent computing optimization problems in other fields.
